# Loading Imatinib inside targeted nanoparticles to prevent Bronchiolitis Obliterans Syndrome

**DOI:** 10.1038/s41598-020-77828-y

**Published:** 2020-11-26

**Authors:** Laura Pandolfi, Roberta Fusco, Vanessa Frangipane, Ramona D’Amico, Marco Giustra, Sara Bozzini, Monica Morosini, Maura D’Amato, Emanuela Cova, Giuseppina Ferrario, Patrizia Morbini, Miriam Colombo, Davide Prosperi, Simona Viglio, Davide Piloni, Rosanna Di Paola, Salvatore Cuzzocrea, Federica Meloni

**Affiliations:** 1 Research Laboratory of Lung Diseases, Section of Cell Biology, IRCCS Policlinico San Matteo Foundation, 27100 Pavia, Italy; 2 Department of Chemical, Biological, Pharmaceutical and Environmental Science, University of Messina, 981000 Messina, Italy; 3 NanoBioLab, Department of Biotechnology and Biosciences, University of Milano-Bicocca, 20100 Milano, Italy; 4 Department of Molecular Medicine, Pathology Unit, University of Pavia; IRCCS Foundation Policlinico San Matteo, 27100 Pavia, Italy; 5 Nanomedicine Laboratory, ICS Maugeri S.P.A., 27100 Pavia, Italy; 6 Department of Molecular Medicine, Biochemistry Unit, University of Pavia, 27100 Pavia, Italy; 7 Department of Internal Medicine, Section of Pneumology, University of Pavia, Pavia, Italy; 8 Department of Pharmacological and Physiological Science, Saint Louis University School of Medicine, Saint Louis, MO USA; 9 Department of Respiratory Diseases, IRCCS Policlinico San Matteo Foundation, Pavia, Italy; 10 Department of Internal Medicine, Section of Pneumology, University of Pavia, 27100 Pavia, Italy

**Keywords:** Transplant immunology, Nanomedicine, Drug delivery

## Abstract

Bronchiolitis Obliterans Syndrome seriously reduces long-term survival of lung transplanted patients. Up to now there is no effective therapy once BOS is established. Nanomedicine introduces the possibility to administer drugs locally into lungs increasing drug accumulation in alveola reducing side effects. Imatinib was loaded in gold nanoparticles (GNP) functionalized with antibody against CD44 (GNP-HCIm). Lung fibroblasts (LFs) were derived from bronchoalveolar lavage of BOS patients. GNP-HCIm cytotoxicity was evaluated by MTT assay, apoptosis/necrosis and phosphorylated-cAbl (cAbl-p). Heterotopic tracheal transplantation (HTT) mouse model was used to evaluate the effect of local GNP-HCIm administration by Alzet pump. GNP-HCIm decreased LFs viability compared to Imatinib (44.4 ± 1.8% vs. 91.8 ± 3.2%, *p* < 0.001), inducing higher apoptosis (22.68 ± 4.3% vs. 6.43 ± 0.29; *p* < 0.001) and necrosis (18.65 ± 5.19%; *p* < 0.01). GNP-HCIm reduced cAbl-p (0.41 GNP-HCIm, 0.24 Imatinib vs. to control; *p* < 0.001). GNP-HCIm in HTT mouse model by Alzet pump significantly reduced tracheal lumen obliteration (*p* < 0.05), decreasing apoptosis (*p* < 0.05) and TGF-β-positive signal (*p* < 0.05) in surrounding tissue. GNP-HCIm treatment significantly reduced lymphocytic and neutrophil infiltration and mast cells degranulation (*p* < 0.05). Encapsulation of Imatinib into targeted nanoparticles could be considered a new option to inhibit the onset of allograft rejection acting on BOS specific features.

## Introduction

Bronchiolitis Obliterans Syndrome (BOS) still remains the principal factor limiting long-term lung transplanted (LTx) survival, whatever the initial triggers are (acute cellular or humoral rejection, cytomegalovirus or community acquired viral infection, de novo occurrence of donor or auto-specific antibodies, gastro-esophageal reflux) the final result is a fibrotic obliteration of small airways, with poor therapeutic options available, besides retransplantation. In the last period, nanomedicine has proven to be a great opportunity to administer drugs by local route, to increase drug efficiency and to reduce side effects. In particular, the chance to administer drug-loaded nanoparticles directly into the lungs through inhalation has gained great attention^1,2^. It is a non-invasive delivery approach which obtains a diffuse distribution of properly designed nanovehicles to deep lung districts with high absorption area and might be crucial in chronic lung diseases. In addition, it is possible to decorate nanovehicles with specific molecules to target pathogenic cells and maximize therapeutic drug efficacy^3,4^.

Our research group recently developed gold nanoparticles (GNP) with the aim to deliver drugs inside lungs and specifically target pathogenic lung fibroblasts (LFs) causing BOS. We already demonstrated that decorating GNPs with half chains of anti-CD44 antibodies (GNP-HC), consistently expressed by LFs, specifically target this type of cells isolated from patients affected by Collagen Tissue Disease–associated Interstitial Lung Fibrosis (CTD-ILD)^5^ and BOS^6^. Even if ILD and BOS have two different underlying causes, in both pathologies the results of inflammatory insults lead to an over-proliferation of LFs and accumulation of extracellular matrix. Moreover, ILD and BOS have in common the limited knowledge about the exact molecular mechanisms underlying the origin of LFs and the absence of an effective therapy. Thus, we decided to study the toxic efficacy of delivering Imatinib (GNP-HCIm), a tyrosine kinase inhibitor with known antifibrotic properties^7,8^, through tracheal instillation in a mouse model of bleomycin-induced pulmonary fibrosis, demonstrating a significant reduction of the fibrotic lesions and macrophages activity modulation^5^. Given these encouraging results, here, we wanted to assess the efficacy of delivering locally Imatinib by GNP-HC in a mouse model of BOS, since the use of a nanovehicle could increase lung deposition of drug limiting extrapulmonary side effects.

As mouse model of BOS, we chose heterotopic tracheal transplantation (HTT) model, commonly accepted as a simple and reproducible surrogate model of airway fibrous obliteration due to allospecific injuries^9^. To mimic local administration, GNP-HCIm have been regularly administered through implanted Alzet pump directly into transplanted tracheal lumen, providing a demonstration that GNP-HCIm are able to prevent tracheal obliteration.

## Results

### In vitro experiments

**Figure 1:**
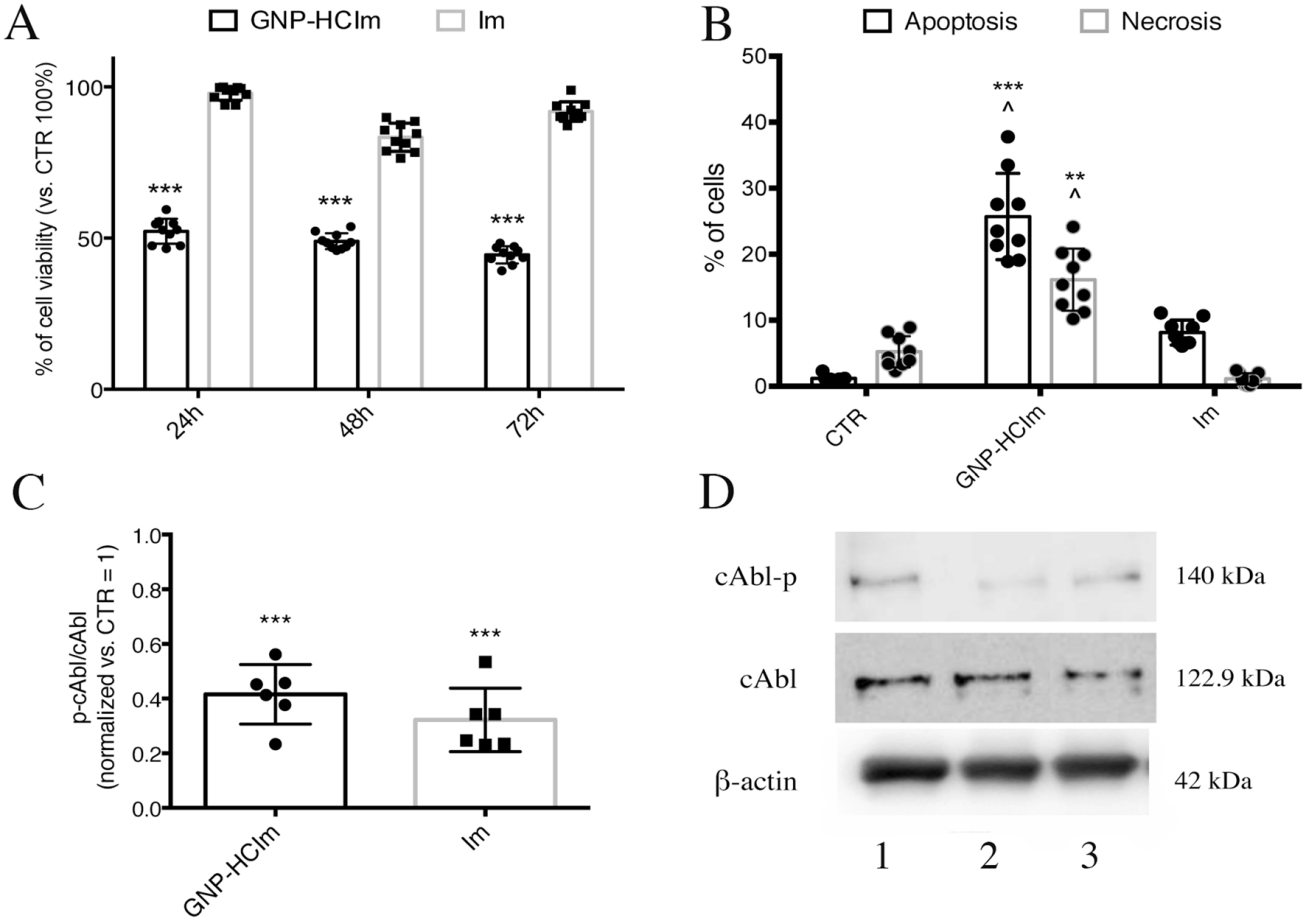
Cytotoxic activity of GNP-HCIm and Imatinib (Im) alone on BOS-derived LFs. (**A**) Cell viability assay after 24, 48 and 72 h of treatment with GNP-HCIm and Im alone at the same concentration (10 μM). Data of two independent replicates (N = 10 for each condition) are represented as mean ± SD. (**B**) Quantification of apoptotic and necrotic cells after 48 h of treatment with GNP-HCIm and Im alone. Apoptotic cells were labeled with PE-Annexin V and necrotic cells with 7-AAD. Data of three independent replicates (N = 9 for each condition) are represented as mean ± SD. (**C**) Semiquantitative analysis of immunoblot of cAbl activity in BOS-derived cells after treatment with GNP-HCIm and Im alone for 24 h. Activity of cAbl was assessed by the quantification of phosphorylated protein related to total cAbl (cAbl-p/cAbl) normalizing results obtained after treatments with CTR cells. (**D**) representative immunoblot using antibodies specific for cAbl-p, or c-Abl or β-actin. Line 1 = CTR; line 2 = GNP-HCIm; line 3 = Im alone. Data of two independent replicates (N = 6 for each condition) are represented as mean ± SD. All graphs are made by Graphpad Prism 6.0; (https://www.graphpad.com/scientific-software/prism/). All data were analyzed by one-way ANOVA followed by a Tukey *post-hoc* test for multiple comparisons. ***, *p* < 0.001 vs. CTR; **, *p* < 0.01 vs. CTR; ^, *p* < 0.001 vs. Im.

Firstly, by confocal microscopy and flow cytometry, we assessed the targeting efficiency of GNP-HC (Figs. S1 and S2) onto LFs derived from BOS-affected patients and on 16HBE cell line (CD44-negative cells), using as control GNP-IgG, confirming data already reported^5,6^. Observing no interaction between GNP-HC and CD44-negative cells we decided to assess the in vitro effect only for GNP-HCIm onto LFs by cytotoxicity assays (MTT), comparing results with Imatinib alone at the same concentration (10 μM). The encapsulation of Imatinib inside GNP increased drug toxicity already after 24 h (51.04 ± 1.79%) and up to 72 h (44.4 ± 1.8%) (Fig. 1A). Imatinib alone showed a time-dependent efficacy but lower than that of GNP-HCIm up to 72 h (91.8 ± 3.2%). To further study if the observed decrease in cell viability was due to the induction of cell death, we incubated LFs with both treatments and assessed the apoptotic and necrotic cell rate by PE-Annexin-V (apoptosis) and 7-AAD (necrosis) labeling. Analyzing cells with flow cytometry, we demonstrated that GNP-HCIm significantly decreased cell viability with respect to control cells, leading to cell death, with 22.68 ± 4.39% apoptotic and 18.65 ± 5.19% necrotic cells. Imatinib alone only resulted in a higher proportion of apoptotic cells (6.43 ± 0.29%) as compared to control.

Lastly, because one of the main targets of Imatinib is cAbl, a tyrosine kinase demonstrated to be involved in modulation of profibrotic cytokine signaling^10^, we wanted to understand if encapsulation of Imatinib inside GNP-HC maintained the same specific molecular activity. After incubating BOS-derived LFs with GNP-HCIm for 24 h, we compared cAbl phosphorylation levels with cells incubated with Imatinib alone. Figure 1C,D show that both treatments led to a significant decrease of protein phosphorylation with respect to control (0.41 GNP-HCIm and 0.24 Imatinib *vs.* CTR) (Fig. S4).

### GNP-HCIm effect on HTT model

**Figure 2:**
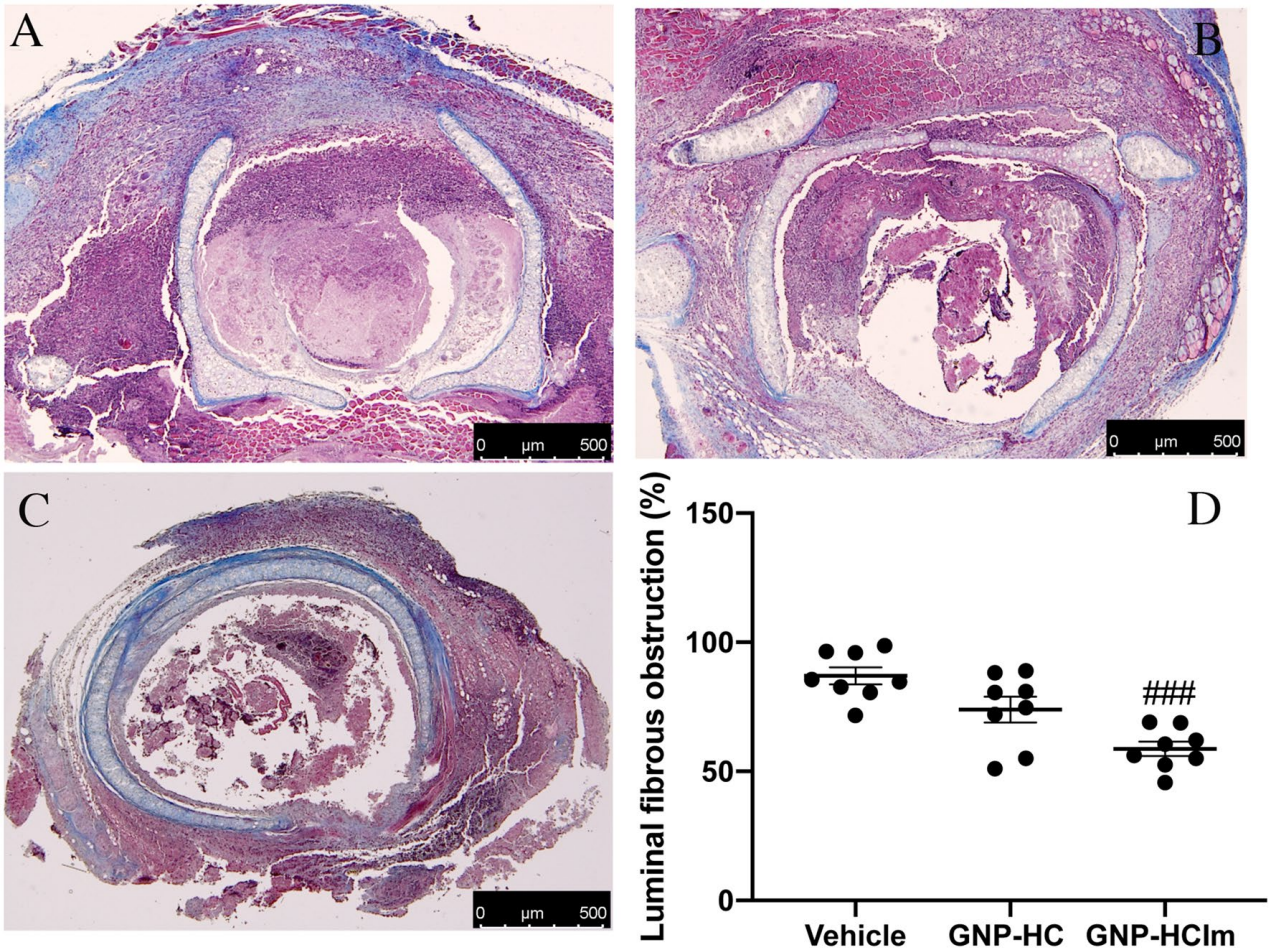
(**A**–**C**) Masson trichrome staining of (**A**) vehicle, (**B**) GNP-HC and (**C**) GNP-HCIm treatments. (**D**) Quantification of collagen area after all treatments. Graph was done by Graphpad Prism 6.0; (https://www.graphpad.com/scientific-software/prism/). Data were represented as mean (N = 8 for each group) ± SEM and analyzed by one-way ANOVA followed by a Bonferroni *post-hoc* test for multiple comparisons. ###*p* < 0.05. Scale bar = 500 μm.

The in vivo toxic effect of GNP-HCIm was studied using the HTT BOS mouse model. In this model, transplanted tracheal segments undergo lymphocytic and neutrophilic infiltration and apoptosis of the airway epithelium, followed by total obliteration of the tracheal lumen due to migration and proliferation of fibroblasts (Fig. 2A, S5A)^9^. After 28 days of treatment with GNP-HCIm directly administered into trachea through Alzet pump, grafts showed a significant reduction of obliterated area (Fig. S5C,D) and collagen deposition (Fig. 2C,D) compared to vehicle (Figs. S5A,D; 2A,D) and to GNP-HC treatment (Figs. S5B,D, 2B,D).

**Figure 3:**
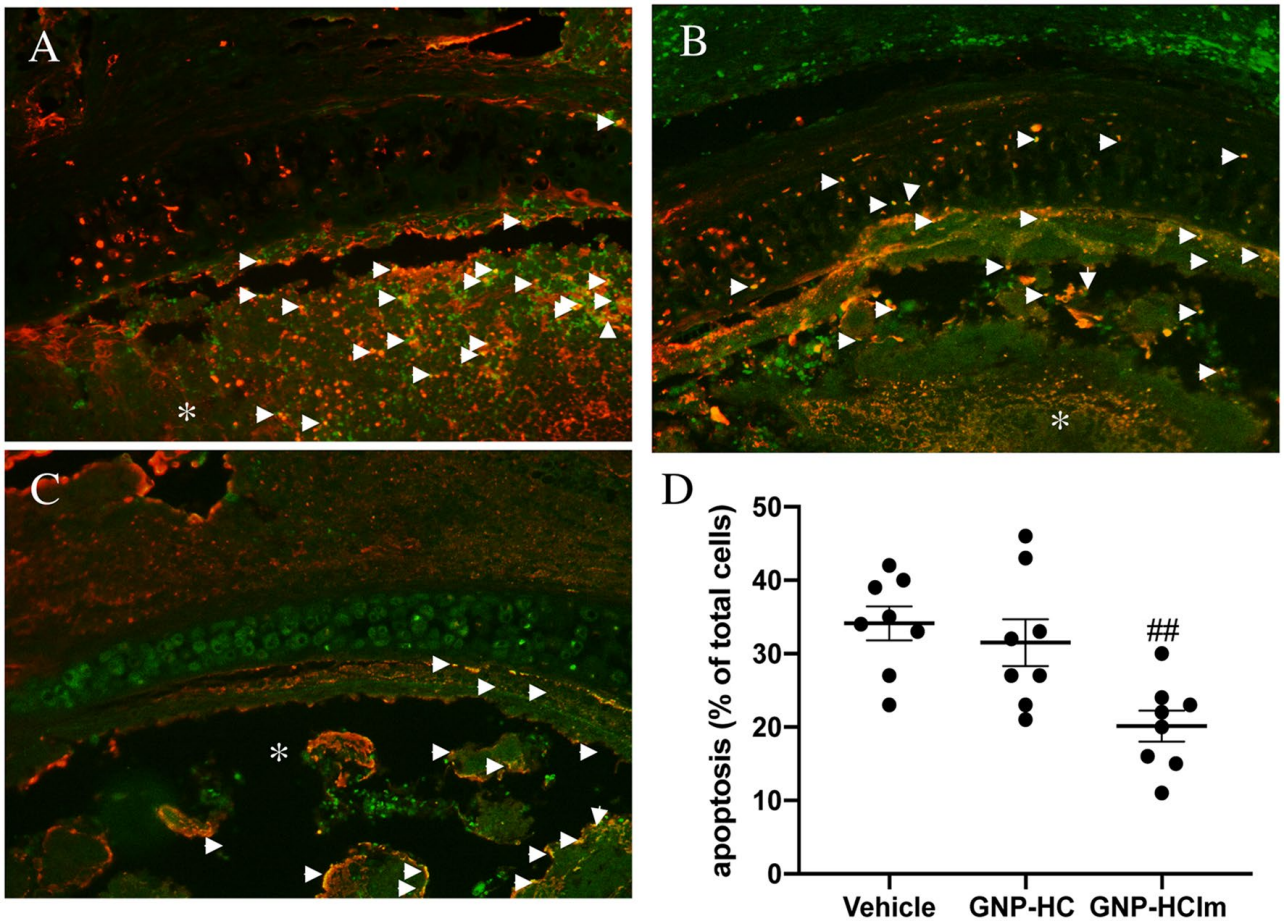
Representative TUNEL assay to visualize the apoptotic rate (red signal; arrows indicate positive cells) of tracheal sections after all treatments. (**A**) Vehicle control sample, (**B**) GNP-HC and (**C**) GNP-HCIm. (**D**) Graphical quantification of TUNEL staining. Graph was done by Graphpad Prism 6.0; (https://www.graphpad.com/scientific-software/prism/). Asterisks = lumen of trachea. 20 × magnification is shown. Data were represented as mean (N = 8 for each group) ± SEM and analyzed by one-way ANOVA followed by a Bonferroni *post-hoc* test for multiple comparisons. ###*p*  < 0.05. Scale bar = 100 µm.

Moreover, the analyses of GNP-HCIm-treated graft sections (Fig. 3C,D) by TUNEL assay showed a lower rate of apoptotic cells compared to vehicle controls (Fig. 3A,D) and GNP-HC (Fig. 3B,D). We observed a concentrated TUNEL signal only in the remaining fibrotic lesions (arrow in Fig. 3C).

### Effect on immune cells

**Figure 4:**
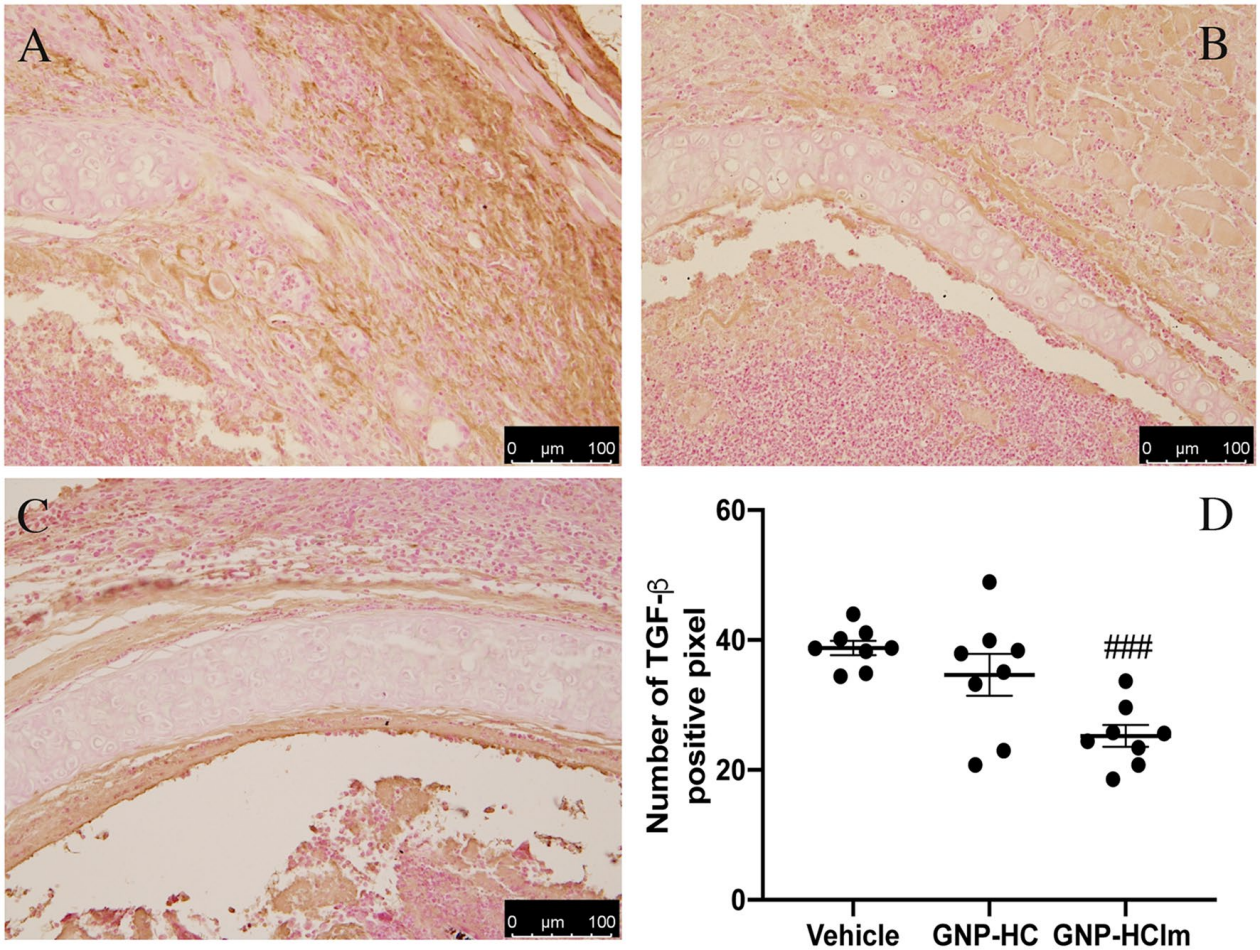
Representative images of explanted trachea sections after (**A**) vehicle, (**B**) GNP-HC and (**C**) GNP-HCIm stained with antibody against TGF-β (brown signal). (**D**) Quantification of observed TGF-β-positive signal. Graph was done by Graphpad Prism 6.0; (https://www.graphpad.com/scientific-software/prism/). Data were represented as mean (N = 8 for each group) ± SEM and analyzed by one-way ANOVA followed by a Bonferroni *post-hoc* test for multiple comparisons. ###*p*  < 0.05. Scale bar = 100 μm.

Considering that one of the most important key players in BOS is TGF-β, we decided to quantify this cytokine in graft samples. By immunohistochemistry, we assessed the surface of TGF-β-positive tissue after all treatments observing a significant decrease of TGF-β signal after 28-days of GNP-HCIm treatment (Fig. 4C,D) as compared to others (Fig. 4A,B,D).

Finally, we examined the effect of treatments on immune cells infiltration, given the important role of innate and adaptive immunity in all BOS phases. In particular, we assessed the quantity of degranulating mast cells, the presence of infiltrating neutrophils and mature lymphocytes. The analyses of GNP-HCIm treated graft sections (Fig. S6C and D) by toluidine blue staining showed a significantly lower number of mast cells as compared to vehicle control (Fig. S6A and D) and GNP-HC (Fig. S6B and D). Regarding neutrophil infiltration, as expected, vehicle controls (Fig. S6E) exhibited an elevated accumulation of neutrophils, as assessed by MPO expression. A high number of MPO-expressing cells was observed in GNP-HC (Fig. S6F), which was significantly reduced in GNP-HCIm treated graft sections (Fig. S6G).

**Figure 5:**
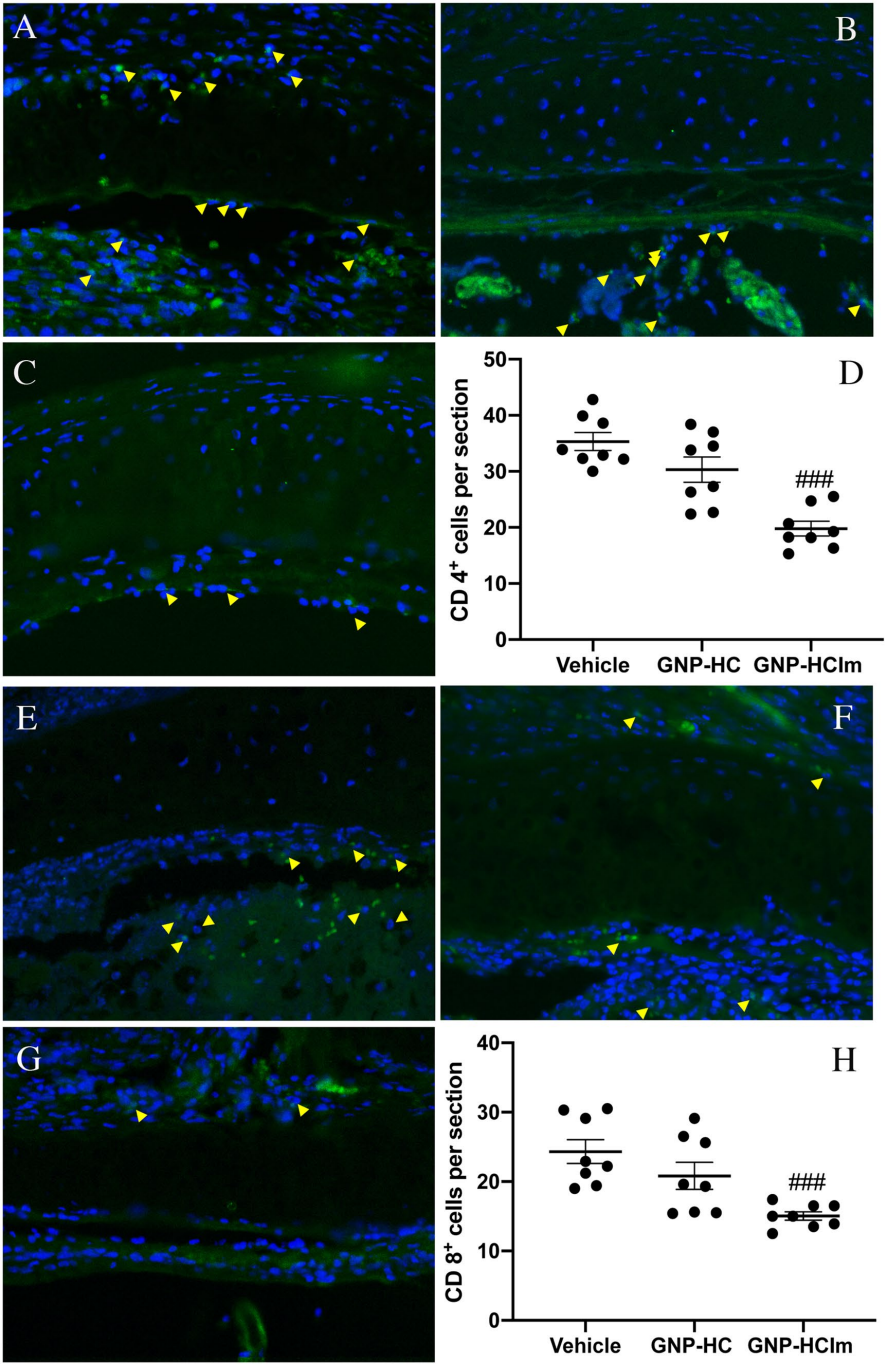
(**A**–**C**) Representative images of explanted trachea sections after (**A**) vehicle, (**B**) GNP-HC and (**C**) GNP-HCIm stained with antibody against CD4. (**D**) Graphical quantification. (**E**–**G**) Representative images of explanted trachea sections after (**E**) vehicle, (**F**) GNP-HC and (**G**) GNP-HCIm stained with antibody against CD8. (**H**) Graphical quantification. For CD4 and CD8 staining, a 20 × magnification is shown. Graphs were done by Graphpad Prism 6.0; (https://www.graphpad.com/scientific-software/prism/). Data were represented as mean (N = 8 for each group) ± SEM and analyzed by one-way ANOVA followed by a Bonferroni *post-hoc* test for multiple comparisons. ###*p*  < 0.05. Scale bar = 100 µm.

At last, immunofluorescence analysis of GNP-HCIm-treated graft sections (Fig. 5C,E) for mature lymphocytes (anti-CD4 and anti-CD8) showed significantly less CD4 + and CD8 + positive cells as compared to vehicle control (Fig. 5A,D,E,H) and GNP-HC (Fig. 5B,D,F,H).

## Discussion

The present results show the feasibility of our nanotechnology-based approach in reducing obliteration occurring in BOS. In particular, we showed the efficacy of CD44-functionalized GNP loaded with Imatinib firstly in vitro and then in vivo in the mouse HTT, commonly accepted as a simple and reproducible surrogate model of airway fibrous obliteration due to allospecific injuries^9^. Several research groups have addressed the potentiality of the local administration of nanoparticles inside lungs, due to lower off-target exposure and the possibility to maximize drug concentration in the target site^11,12^. However, the targeting of aberrant cells is a crucial point, also when dealing with the inhalatory route, in order to spare unwanted injury to normal epithelial cells. For this reason, we decided to functionalize GNP with the half chain of an anti-human antibody directed towards CD44, a glycoprotein expressed at high rate by (myo-)fibroblasts that are the main players of bronchiolar obliteration in BOS patients. We already demonstrated that this surface functionalization approach is efficient and neither alters deep lung microenvironment nor elicits a local inflammatory reaction^5,6,13^.

In the present study, we analyzed the efficacy of Imatinib-loaded CD44 targeted nanovehicles both in vitro and in vivo. The choice to use Imatinib as a drug to be encapsulated into GNPs was based on different reasons. (a) It has been proven that Imatinib is a potent anti-fibrotic drug through the inhibition of cAbl leading to a reduction of cell proliferation, anti-apoptotic signaling and migration^7,8,10,14^. (b) Imatinib antifibrotic effect has already been demonstrated on the HTT model administered daily by intraperitoneal route^8^. (c) Recently, Imatinib has been reported to stabilize lung function in children with BOS after allogenic blood and marrow transplantation, but this treatment has been associated to important side effects such as cytopenia and fluid retention^15–17^. Thus, it can envisage that the addition of this drug to the current immunosuppressive regimen of a lung recipient with BOS might be associated to a consistent toxicity. These considerations prompted out effort to develop a new Imatinib delivery approach exploiting nanotechnology to allow local administration and reduce side effects. By in vitro experiments we demonstrated that the encapsulation of Imatinib into targeted GNPs increased the toxic effect of the drug on LFs derived from patients affected by BOS, thanks to a higher and longer intracellular release which increase drug effect, confirming results obtained for CTD-ILD^5^. Moreover, we proved that cell viability decrease was due to a significantly higher induction of apoptosis than that induced by free drug at the same concentration (Fig. 1A,B). This result is in agreement with the observation of reduced phosphorylated cAbl, since its inhibition is known to have a pro-apoptotic effect. In fact, western blot analyses of cells treated with GNP-HCIm confirmed that Imatinib release from GNP was able to reduce phosphorylation of cAbl, the post-translational modification related to its tyrosine kinase activity (Fig. 1C).

Based on in vitro evidences, we moved to test our nanovehicle in the most common BOS animal model: the HTT mouse model. To mimic the local delivery, we adapted our model with an Alzet pump inserted subcutaneously between the mice scapulae, nearby the tracheal graft. This method allowed us to administer a GNP-HCIm solution with a constant flow juxtaposed to tracheal lumen mimicking a timeline drug administration. Analyzing transplanted trachea sections after treatment with GNP-HCIm, we had the confirmation that local administration approach efficiently prevented occlusion of tracheal lumen compared to vehicle treated transplanted trachea and GNP-HC (Fig. 2). Furthermore, we found a significant reduction of the apoptotic rate in the surrounding epithelium (Fig. 3). This is relevant since epithelial damage is one of the most crucial insults to epithelial mesenchymal transition (EMT)^18^, the precursor step of tracheal fibroobliteration^19^. In the same context, the cytokine TGF-β has been found to have pleiotropic immunoregulatory properties: it plays a central role in EMT induction, it is a critical mediator of extracellular remodeling and fibroproliferation and it is involved in the pathogenesis of a wide variety of disease states. The profibrotic effects of TGF-β are driven by binding and activation of the TGF-β receptor complex, which promotes synthesis of procollagens, the precursors to mature collagen^20^. Interestingly, in Fig. 4 it is possible to observe that our treatment decreased significantly the percentage of TGF-β-positive cells in the treated tissue not limited to the tracheal lumen, but more importantly in the surrounding tissues. We have already reported that Imatinib-loaded GNPs, after inhalation, are entrapped inside macrophages, leading to a modulation of macrophage activity reducing their pro-fibrogenic M2 phenotype and also decreasing the release of IL-8, a pro-inflammatory cytokine^5^. Here, we confirm and extend our observations suggesting that treatment with our nanovehicle is able to decrease both pro-inflammatory and pro-fibrotic signals. Beside the decrease of TGF-β signal in treated mice (Fig. 4) we detected a decrease of specific cell infiltrates in treated graft sections. As for lymphocytes, which have been shown to play a relevant role in HTT model triggering allospecific injury^9^, we observed high lymphocytic infiltration in untreated graft sections (Fig. 5A), and a significant reduction after GNP-HCIm treatment (Fig. 5C). The same result was obtained with the infiltration of neutrophils (Fig. S6E-G) that are known to be one of the major effectors of BOS^21,22^.

Finally, mast cells have been demonstrated to play a pathological role in many infiltrative fibrotic lung diseases. They release a variety of cytokines, lipid-derived mediators, amines, proteases, and proteoglycans, all of which can regulate adjacent cells and the metabolism of the extracellular matrix of connective tissue. Moreover, they are physiologically abundant in donor lung airway walls and alveolar interstitium, but after transplantation their numbers could increase in association with acute rejection and BOS^23,24^. In fact, we observed high mast cells numbers in heterotopic tracheal allograft sections treated with vehicle (Fig. S6A), that decreased with GNP-HCIm treatment (Fig. S6C).

HTT is a reproducible, feasible and fast model of airway obliteration, thus, we decided to adopt it in order to study, for the first time, the effects of drugs/nanovehicles on chronic rejection; however, we are aware about the several limitations that characterize HTT model. For example, HTT is not a vascularized model even if it has been described that in this model angiogenesis occurred from surrounding tissue during the course of wound healing^25^. But more importantly, it does not concern lungs, but tracheal graft^26^. In fact, our future perspectives will be the confirmation of these results in a more complex allograft rejection animal model, such as BOS induced in mice lungs with anti-HLA administration^27^, or by the orthotopic single lung transplantation in mice, which more resembles the human situation compared to other models^28,29^.

Regarding the nature of nanoparticle material, we are already working on more biodegradable nanoparticles^30^, giving the chronic nature of BOS and the consequent need of a chronic inhaled administration. We already demonstrated that a 28-day administration of the same GNP by inhalation in the mouse model of bleomycin-induced pulmonary fibrosis could lead to an accumulation of GNP in alveolar macrophages^5^. So, the next step will be to deliver Imatinib into more biocompatible vehicles in order to avoid accumulation of gold material into the lungs.

In conclusion, the encapsulation of Imatinib inside targeted nanoparticles decreased significantly all the key features of HTT BOS model, from inflammatory cells infiltration to tracheal lumen obliteration, suggesting that could be a promising preventive therapy for LTx patients. Moreover, this paper brings a further pivotal demonstration that nanotechnology represents a very promising option to vehicle locally those drugs that might be difficult to use in chronic lung disorders due to their systemic toxicity.

## Methods

### Ethic approvals

The cells isolation from BAL patients was approved was approved by the IRCCS Policlinico San Matteo ethic committee (prot 20100005334) and all patients gave informed consent in accordance with the Declaration of Helsinki.

All in vivo experiments were approved by University of Messina (prot. 137/2017-pr) and followed the new guidelines and regulations of USA (Animal Welfare Assurance No A5594-01), Europe (EU Directive 2010/63), Italy (D.Lgs 2014/26) and the ARRIVE guidelines.

### Nanoparticles synthesis

GNP-HCIm were synthesized and characterized following the previously reported procedure^5^.

### Cells isolation and culture

LFs were isolated from N = 6 BAL fluids of BOS patients (at different stages of BOS) as previously reported^6^. Isolated LFs were cultured in high glucose Dulbecco’s modified Eagle medium (DMEM) with 10% fetal bovine serum (FBS), 100 U mL^–1^ penicillin/streptomycin (P/S) solution and 100 U mL^–1^ L-glutamine. All in vitro experiments were conducted treating cells with 25 µg mL^-1^ of GNP-IgG or GNP-HCIm, and with 10 µM Im alone. As control, we treated cells also with GNP conjugated with antibody isotype control (GNP-IgG). The concentrations of nanoparticles and Imatinib alone were decided following previous published results^5^. 16-HBE cell line was used as CD44-negative cell line control and cultured in the same medium as LFs.

### Confocal microscopy and flow cytometry

To analyze nanoparticle internalization by confocal microscopy, 1.5 × 10^5^ LFs and 16-HBE were seeded on 35 mm dishes. After 24 h, cells were incubated with Alexa Fluor 488-labeled GNP-HC and GNP-IgG for 2 h at 37 °C. At the end of incubation, cells were washed with Phosphate Buffer Saline (PBS) solution, fixed with 4% paraformaldehyde, and cell nuclei were labeled with 4′,6′-diamidino-2-phenylindole (DAPI). GNPs green signals were observed by confocal laser microscopy (FluoView, FU10i, Olympus).

To quantify GNPs cell internalization, 3 × 10^5^ LFs and 16-HBE were seeded in 12-wells plate and incubated with fluorescent GNP-HC and GNP-IgG for 2 h at 37 °C. After treatments, cells were washed, harvested and analyzed by Navios Flow Cytometer (Beckman Coulter).

### Cell viability and apoptosis/necrosis assays on LFs

MTT (3-(4,5-dimethylthiazol-2-yl)-2,5-diphenyltetrazolium bromide) test (Sigma-Aldrich – Missouri, USA) was used to assay viability of LFs. Briefly, 5 × 10^3^ cells were incubated with 25 µg mL^−1^ of GNP-HCIm and with 10 µM Imatinib for 2 h at 37 °C. Afterwards, fresh medium was added to continue incubation up to 24, 48, and 72 h. Results were expressed as percentage of variation *vs*. untreated LFs (CTR) set to 100%.

Apoptosis and cell death of LFs were assessed by phycoerythrin-labeled annexin V (PE-Annexin V) and 7-AAD (Molecular Probes, Life Technologies) incorporation by flow cytometry at 48 h after 25 µg mL^-1^ GNP-HCIm and 10 µM Imatinib incubation. By flow cytometry, we quantified the percentage of cells positive for PE-Annexin V (apoptotic cells) and for 7-AAD (necrotic cells).

### Western blot analyses

After treating LFs with 25 µg mL^-1^ GNP-HCIm and 10 µM Imatinib for 24 h, cells were lysed. Proteins (10 µg) were loaded onto SDS-PAGE and transferred onto a PVDF membrane using a Trans Blot turbo system (Bio-Rad). After 1 h incubation in 5% Bovine Serum Albumin (BSA) diluted in PBS and three washes with PBS containing 0.1% Tween 20 (TBST), the membrane was incubated overnight with anti-c-Abl (phospho Y412) antibody (ab4717, Abcam) at a 1:1000 dilution in 1% BSA. After three washes with TBST (10 mL), membranes were incubated with Rabbit anti-Mouse IgG H&L (HRP) (ab6728, Abcam) secondary antibody at a 1:2000 dilution in 1% BSA in TBST, for 1 h at room temperature. Clarity Western ECL (Bio-Rad) solution was used according to the provided protocol. The same procedure was applied to identify c-Abl by anti-c-Abl antibody (ab15130, Abcam) at a 1:100 dilution and β-actin (15G5A11/E2, Cat #MA1-140, Thermo Fisher Scientific) at a 1:5000 dilution. Also in this case we used as secondary antibody Rabbit anti-Mouse IgG H&L (HRP) (ab6728, Abcam). All immunoblots were acquired with the ImageQuant LAS 4000 analyzer (GE Healthcare).

### Animals

Pathogen-free, male C57BL/6 and Balb/c mice (Jackson Laboratory) weighing 20**–**24 g were used. All animals were housed in the specific pathogen-free facility and had access to water and food ad libitum. The mice were housed in cages under hygienic conditions and kept at room temperature (19–25 °C) with a 12 h light/12 h dark cycle. Mice were acclimated for 1 week before the experiment.

### Donor and recipient procedures

Tracheas from Balb/c mice were implanted into C57BL/6 mice as previously described^31^. Grafts were harvested on Days 28 after transplantation for histologic and immunohistologic analyses.

### Animal treatment

To treat tracheal transplanted mice, we used Alzet model 2004 mini osmotic pumps (Charles River Laboratories International) to deliver drugs at constant rate. Alzet pump was implanted subcutaneously through a small incision in the skin between the scapulae. Using a hemostat, a small pocket was formed by spreading the subcutaneous connective tissues apart to insert the pump, with the flow moderator pointing away from the incision. The skin incision was closed with sutures. The pumping rate was 1 ulh^−1^ (± 0.15 ulh^−1^), and the reservoir volume was 200 µL. Three groups of treatment were done: vehicle (saline solution), GNP-HCIm and GNP-HC. Nanoparticles were administered immediately after tracheal transplantation with a final concentration of 50 µg mL^−1^.

### Histological examinations of grafts

The grafts were harvested on Day 28 after transplantation for histologic and immunohistochemical analyses. Cross-sectional specimens were fixed in 10% buffered formalin, embedded in paraffin, sectioned at 5 µm-thickness, stained with haematoxylin–eosin (H&E), and Masson’s trichrome. All specimens were examined in blind fashion and scored as previously described^32^. Images were then analyzed with Zeiss AxioVision 4.7 software and surface from the tracheal cartilage and the free lumen were measured. Residual free lumen was calculated as follows: (free lumen/surface at cartilage) × 100. Results are expressed as percentages.

### TUNEL staining

TUNEL staining protocol was performed according to a Roche protocol^32^ unstained paraffin sections of retrieved tracheal grafts. This protocol is used for detection and quantification of apoptosis (programmed cell death) at single cell level, based on labeling of DNA strand breaks.

### Mast cells evaluation

Sections were stained with toluidine blue in order to assess mast cell degranulation. The mast cells count was performed on each slide through a Leica DM6 microscope. The digital images were opened in ImageJ, followed by deconvolution using the color deconvolution plug-in. When the Immunohistochemistry (IHC) Profiler plugin is selected, it mechanically plots a histogram profile of the deconvoluted diaminobenzidine image, and a corresponding scoring log is exhibited. The histogram profile relates to the positive pixel intensity value gotten from a computer program^33^. All immunohistochemical analyses were carried out by 2 observers masked to the treatment.

### Immunohistochemical localization for TGF-β

Unstained paraffin sections were incubated overnight with the antibody anti-TGF-β (1:250, #sc17792, Santa Cruz Biotechnology) dissolved in PBS. Images were collected using a Leica DM6 microscope. For graphic display of densitometric analyses, the % of positive staining (brown staining) was measured by computer-assisted color image analysis. The percentage area of immunoreactivity (determined by the number of positive pixels) was expressed as % of total tissue area (red staining) within five random fields at 40 × magnification.

### Immunofluorescence for CD4, CD8 and Myeloperoxidase (MPO)

Unstained sections were incubated with one of the next primary mouse monoclonal antibodies: anti-CD4 or anti-CD8 (for mature lymphocytes) or MPO (neutrophils) (1:100, Santa Cruz Biotechnology) in a humidified chamber at 37 °C overnight. Sections were washed with PBS and were incubated with secondary antibody with fluorescein isothiocyanate-conjugated anti-mouse Alexa Fluor 488 (1:2000, Molecular Probes, Life Technologies) for 1 h at 37 °C. Sections were rinsed and nuclear counterstain with 2 μg ml^−1^ DAPI in PBS was added. Sections were observed using a Leica DM2000 microscope. Each microscopic field was acquired at 20 × magnification to maximize signal and analysed.

### Statistical analysis

For in vitro analyses, values are shown as mean ± standard deviation (SD). Data were analyzed by one-way ANOVA followed by a Tukey *post-hoc* test for multiple comparisons using Prism software (Graphpad Prism 6.0; https://www.graphpad.com/scientific-software/prism/). A p-value < 0.05 has been considered significant. For in vivo analyses all values are showed as mean ± standard error of the mean (SEM) of N observations. N denotes the number of animals employed. In the experiments including immunohistochemistry or histology, at least three experiments were performed on different experimental days. Data were analyzed by one-way ANOVA followed by a Bonferroni *post-hoc* test for multiple comparisons. A *p* value < 0.05 was considered significant. The number of animals used for in vivo studies was carried out by G * Power 3.

## Supplementary information

Supplementary Figures.
