# Supplementary material for: Loading Imatinib inside targeted nanoparticles to prevent Bronchiolitis Obliterans Syndrome
Source: Sci Rep. 2020 Nov 26;10:20726. doi: 10.1038/s41598-020-77828-y (PMC7693282; doi:10.1038/s41598-020-77828-y)
Supplement: Supplementary file 1 — Supplementary Figures. [file 41598_2020_77828_MOESM1_ESM.docx]

LOADING IMATINIB INSIDE TARGETED NANOPARTICLES TO PREVENT BRONCHIOLITIS OBLITERANS SYNDROME

Laura Pandolfi^1*^, Roberta Fusco^2^, Vanessa Frangipane^1^, Ramona D’Amico^2^, Marco Giustra^3^, Sara Bozzini^1^, Monica Morosini^1^, Maura D’Amato^1^, Emanuela Cova^4^, Giuseppina Ferrario^4^, Patrizia Morbini^4^, Miriam Colombo^3^, Davide Prosperi^3,5^, Simona Viglio^6^, Davide Piloni^7^, Rosanna Di Paola^2^, Salvatore Cuzzocrea^2,8^ and Federica Meloni^9^


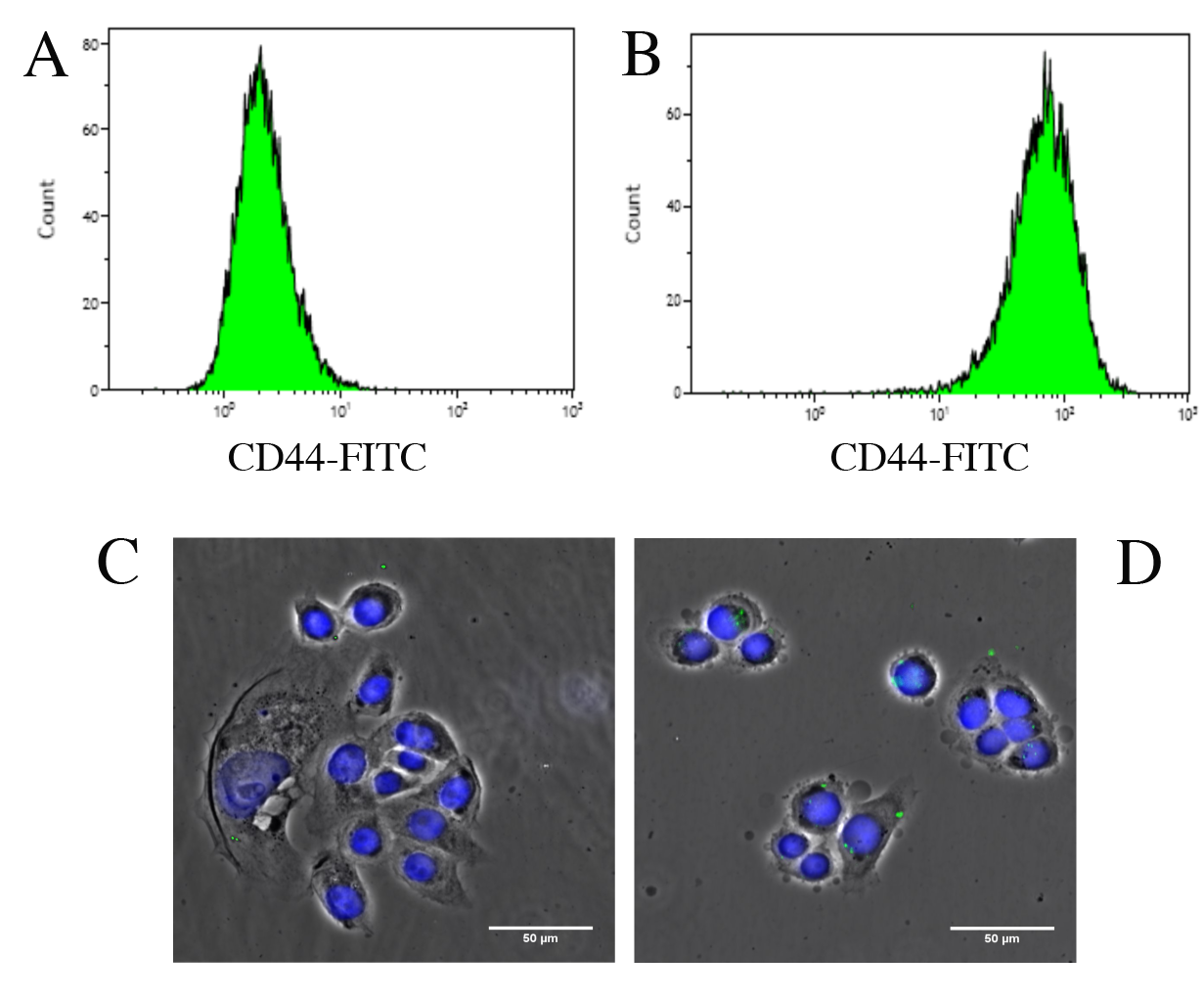


Figure S1. (A,B) Analyses of CD44 by flow cytometry of (A) 16-HBE and (B) LFs derived from BOS-affected patients. (C,D) Confocal images of 16-HBE treated with (C) GNP-IgG and (D) GNP-HC for 4 h at 37 °C. Scale bar = 50 μm


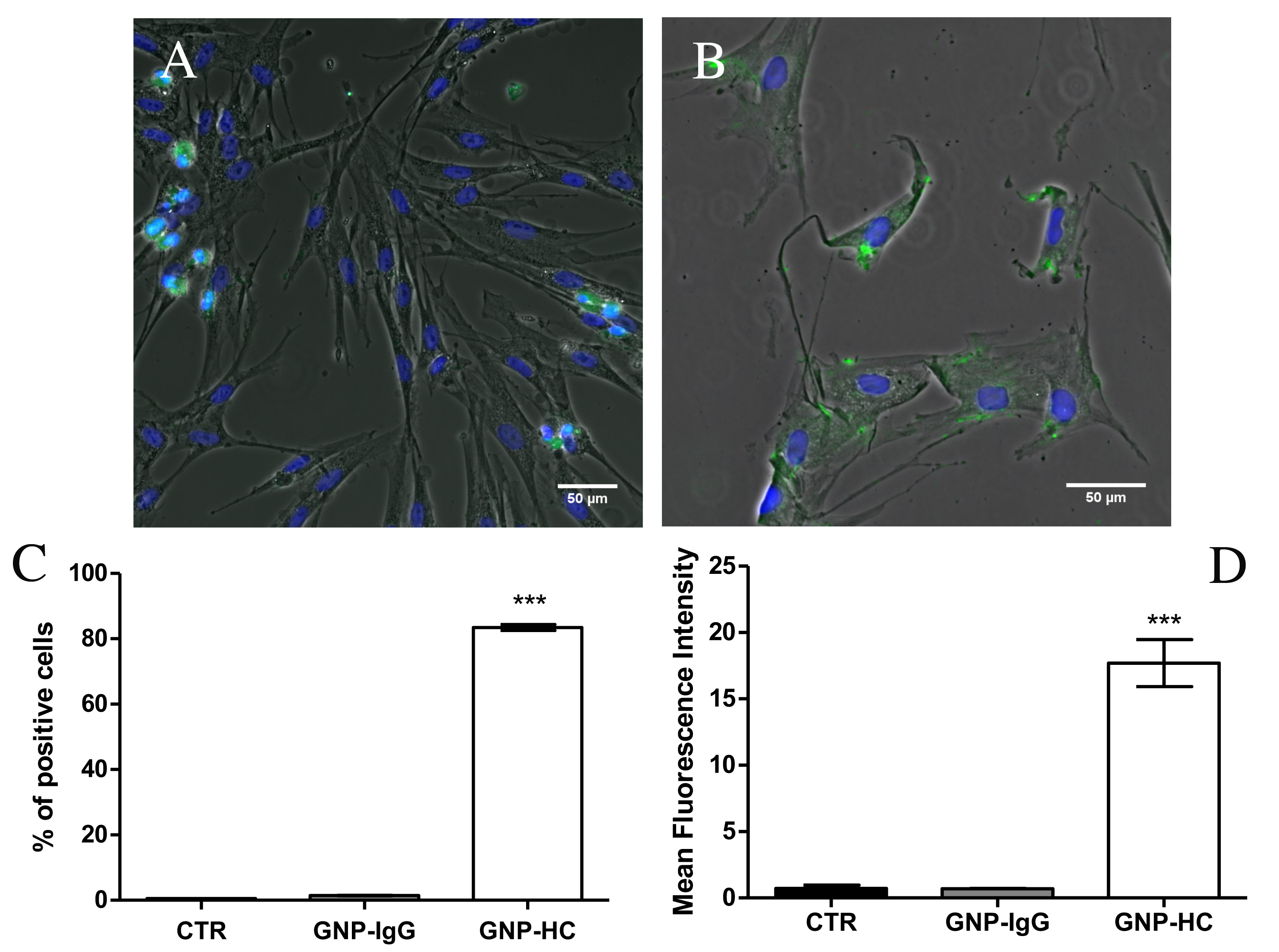


Figure S2. Analyses of GNP internalization in BOS-derived cells. A,B) Confocal images of BOS treated with (A) GNP-IgG and (B) GNP-HC for 4 h. C,D) Flow cytometry quantification of GNP-IgG and -HC uptake in BOS-derived cells. C) percentage of cells positive for Alexa Fluor 488 signal. Data are represented ad mean ± standard deviation. ***, p < 0.001 vs. CTR


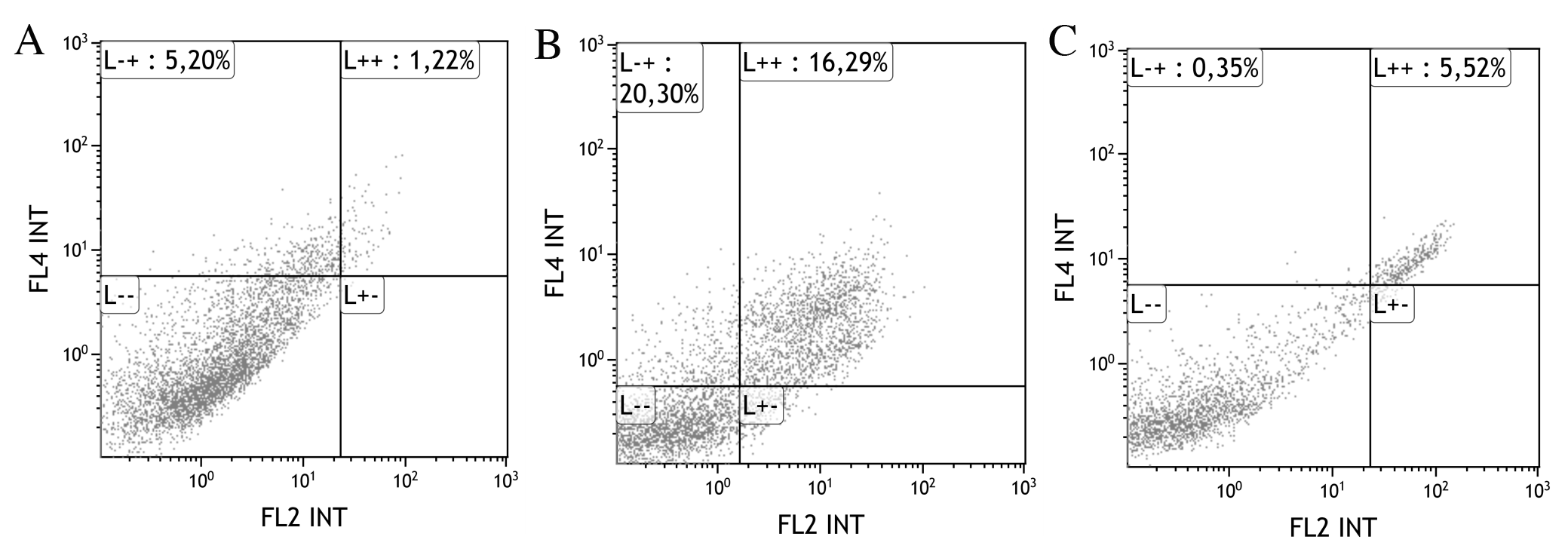


Figure S3. Representative FACS plots of CTR (A), GNP-HCIm (B) and Im alone (C) after 48h of treatment. FL2 = Annexin signal; FL4 = 7AAD signal.


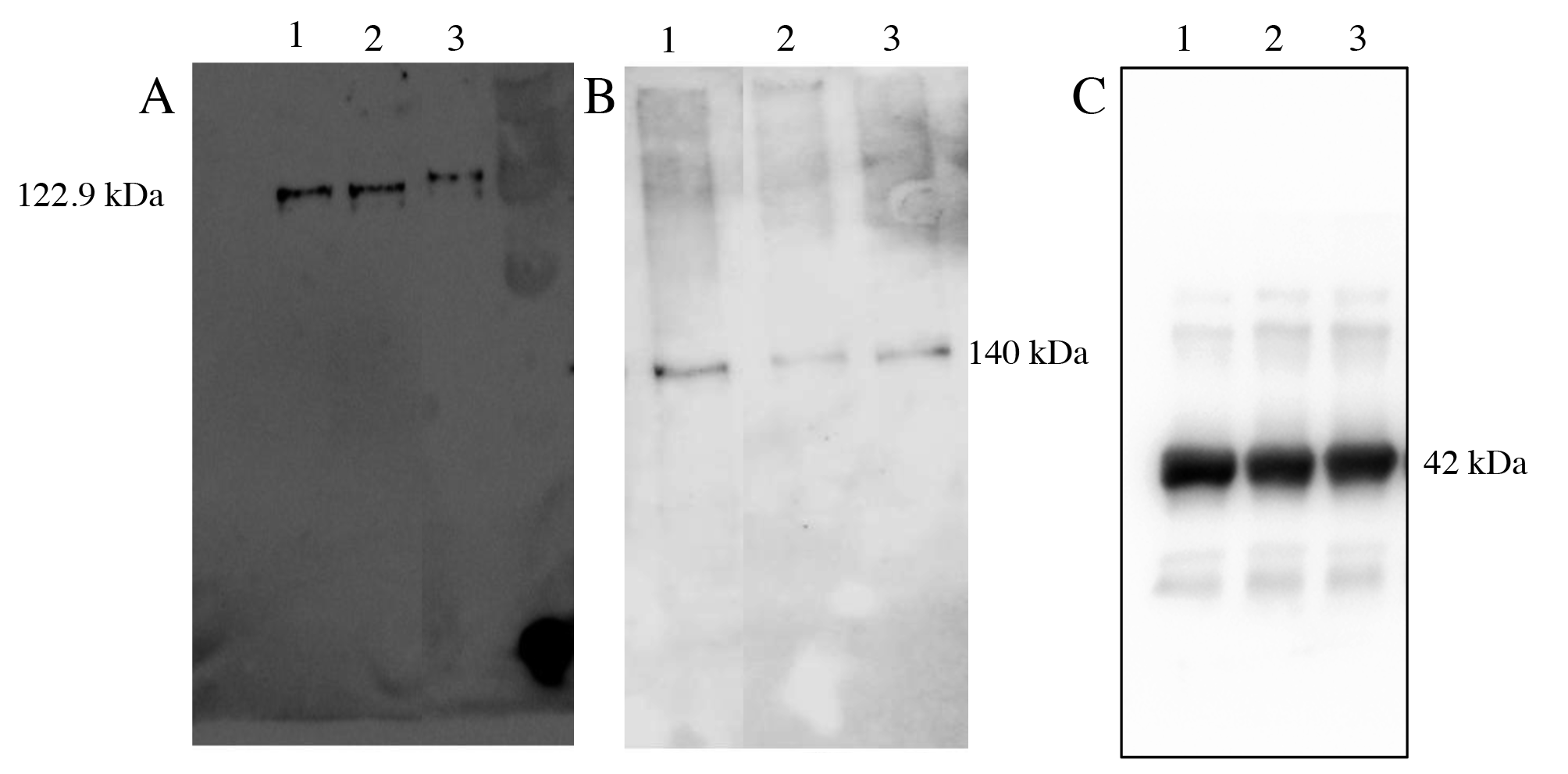


Figure S4. Representative blot for immunodecorated with antibodies against (A) c-Abl, (B) phospho-c-Abl and (C) β-actin. Lane 1 = CTR; lane 2 = GNP-HCIm; lane 3 = Im alone. The images are cropped because we loaded other experiments in the same gel.


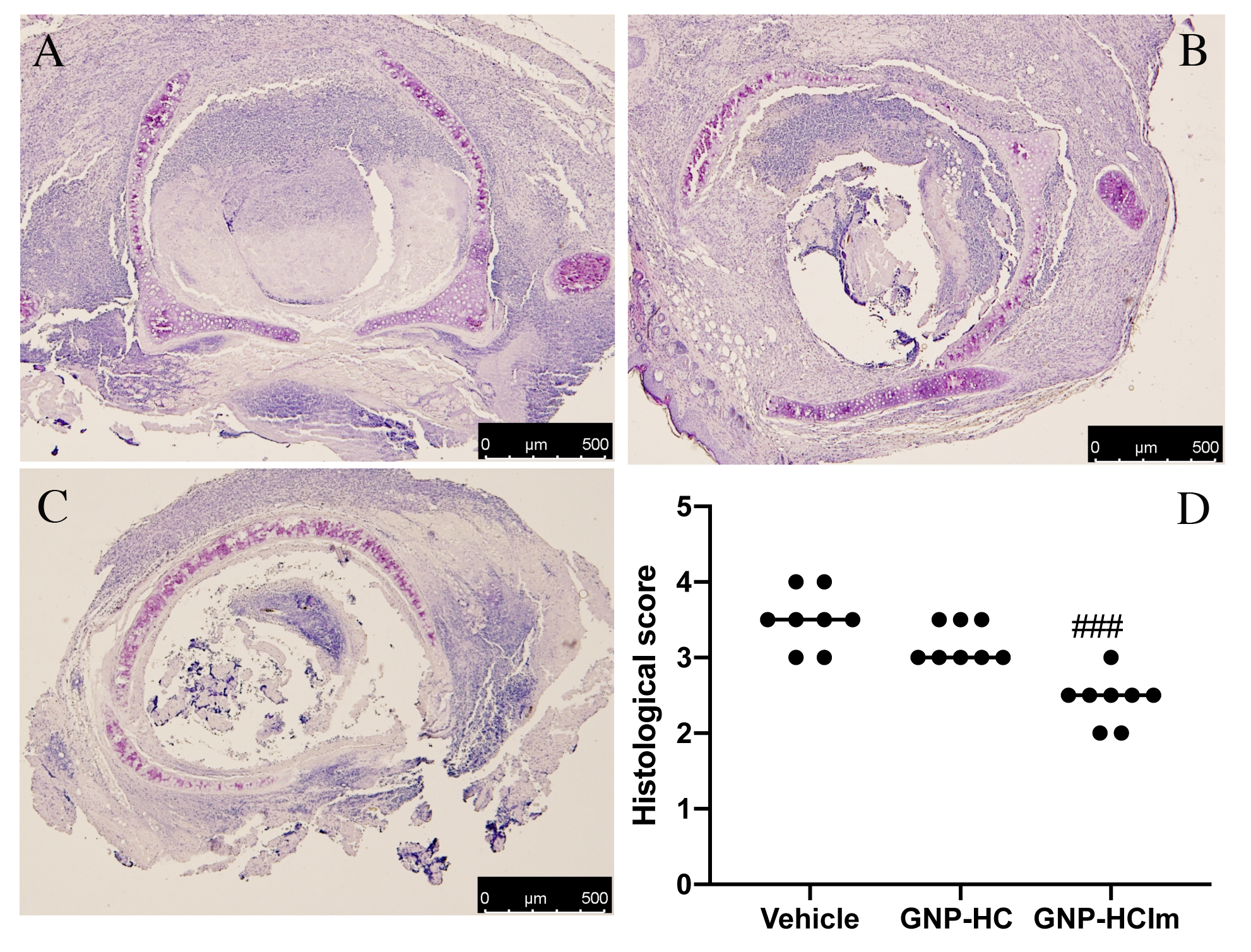


Figure S5. (A-C) H&E staining of tracheal graft sections after (A) vehicle, (B) GNP-HC and (C) GNP-HCIm treatments. (D) Quantification of obliterated area after all treatments. Data were represented as mean (N = 8 for each group) and analyzed by one-way ANOVA followed by a Bonferroni *post-hoc* test for multiple comparisons. ###p < 0.05. Scale bar = 500 μm


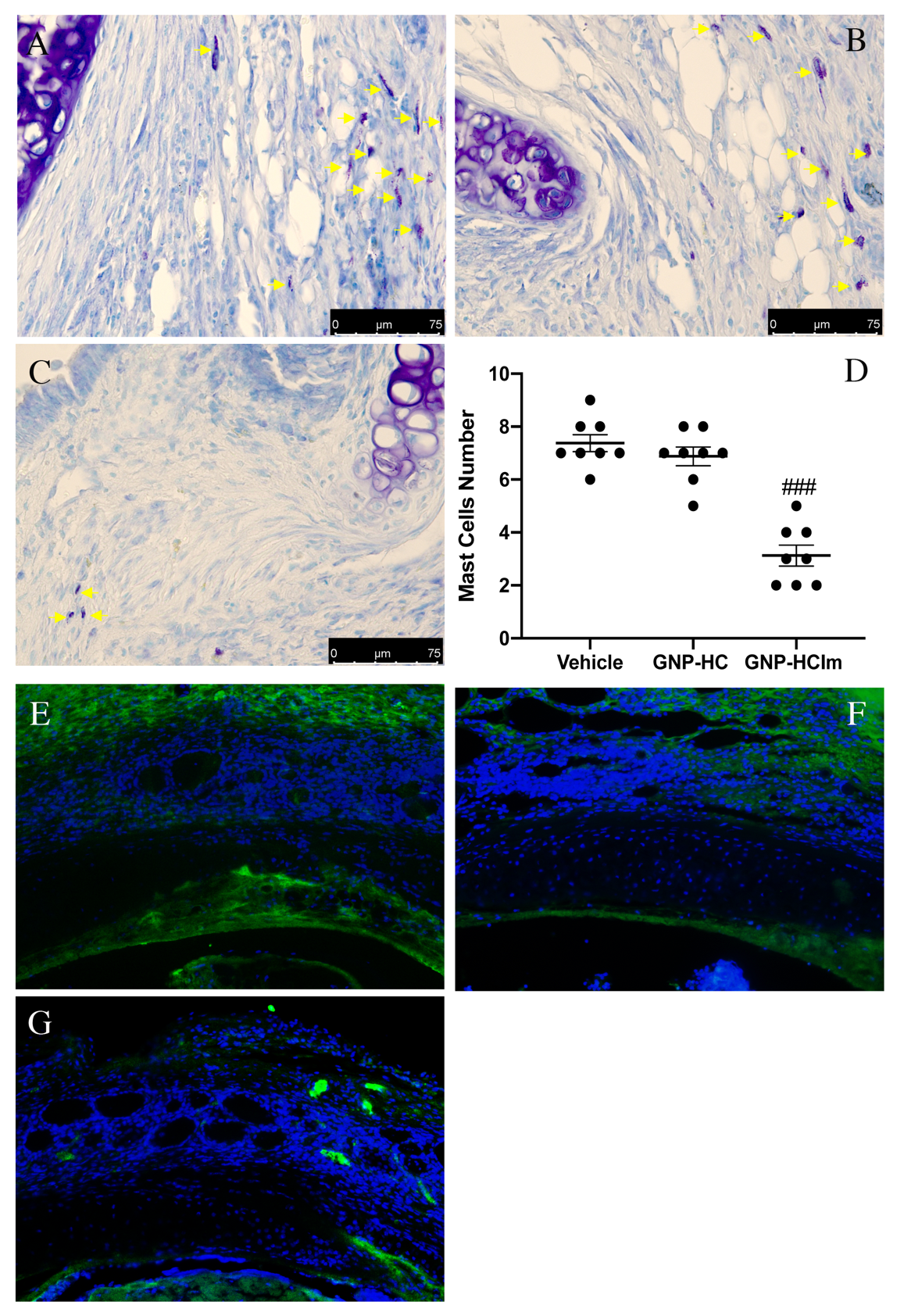


Figure S6. (A-C) Representative images of explanted trachea sections after (A) vehicle, (B) GNP-HC and (C) GNP-HCIm stained with Toluidine blue. (D) Quantification of mast cell number. Data were represented as mean (N = 8 for each group) and analyzed by one-way ANOVA followed by a Bonferroni *post-hoc* test for multiple comparisons. ###p < 0.05. Scale bar = 75 μm. (E-G) Representative images of explanted trachea sections after (E) vehicle, (F) GNP-HC and (G) GNP-HCIm stained with antibody against MPO (green signal).
